# Cohort profile: King’s Health Partners bladder cancer biobank

**DOI:** 10.1186/s12885-020-07437-5

**Published:** 2020-09-25

**Authors:** Pinky Kotecha, Charlotte L. Moss, Deborah Enting, Cheryl Gillett, Magdalene Joseph, Debra Josephs, Sarah Rudman, Simon Hughes, Fidelma Cahill, Harriet Wylie, Anna Haire, James Rosekilly, Muhammad Shamin Khan, Rajesh Nair, Ramesh Thurairaja, Sachin Malde, Mieke Van Hemelrijck

**Affiliations:** 1 Translational Oncology & Urology Research (TOUR), School of Cancer and Pharmaceutical Sciences, King’s College London, Guy’s Hospital, 3rd Floor Bermondsey Wing, London, SE1 9RT UK; 2 Department of Oncology, Guy’s and St Thomas’ NHS Foundation Trust, London, UK; 3 King’s Health Partners Cancer Biobank, School of Cancer and Pharmaceutical Sciences, King’s College London, London, UK; 4 Department of Urology, Guy’s and St Thomas’ NHS Foundation Trust, London, UK

**Keywords:** Bladder cancer, Real world evidence, Biobank

## Abstract

**Background:**

Bladder cancer (BC) is the 9th most common cancer worldwide, but little progress has been made in improving patient outcomes over the last 25 years. The King’s Health Partners (KHP) BC biobank was established to study unanswered, clinically relevant BC research questions.

Donors are recruited from the Urology or Oncology departments of Guy’s Hospital (UK) and can be approached for consent at any point during their treatment pathway.

At present, patients with bladder cancer are approached to provide their consent to provide blood, urine and bladder tissue. They also give access to medical records and linkage of relevant clinical and pathological data across the course of their disease. Between June 2017 and June 2019, 531 out of 997 BC patients (53.3%) gave consent to donate samples and data to the Biobank. During this period, the Biobank collected fresh frozen tumour samples from 90/178 surgical procedures (of which 73 were biopsies) and had access to fixed, paraffin embedded samples from all patients who gave consent. Blood and urine samples have been collected from 38 patients, all of which were processed into component derivatives within 1 to 2 h of collection. This equates to 193 peripheral blood mononuclear cell vials; 238 plasma vials, 224 serum vials, 414 urine supernatant vials and 104 urine cell pellets. This biobank population is demographically and clinically representative of the KHP catchment area.

**Conclusion:**

The King’s Health Partners BC Biobank has assembled a rich data and tissue repository which is clinically and demographically representative of the local South East London BC population, making it a valuable resource for future BC research.

## Background

Bladder cancer is the 9th most common cancer worldwide with around 430,000 new cases diagnosed in 2012 [1]. Nevertheless, outcomes in bladder cancer have remained largely unchanged over the last 25 years and much remains unknown about this complex disease, especially with regard to disease aetiology and progression [2]. Urological biobanking can therefore provide a valuable resource to answer a number of clinically relevant research questions [2], spurring translation into practical applications and ultimately leading to improvements in public health and healthcare.

**Fig. 1:**
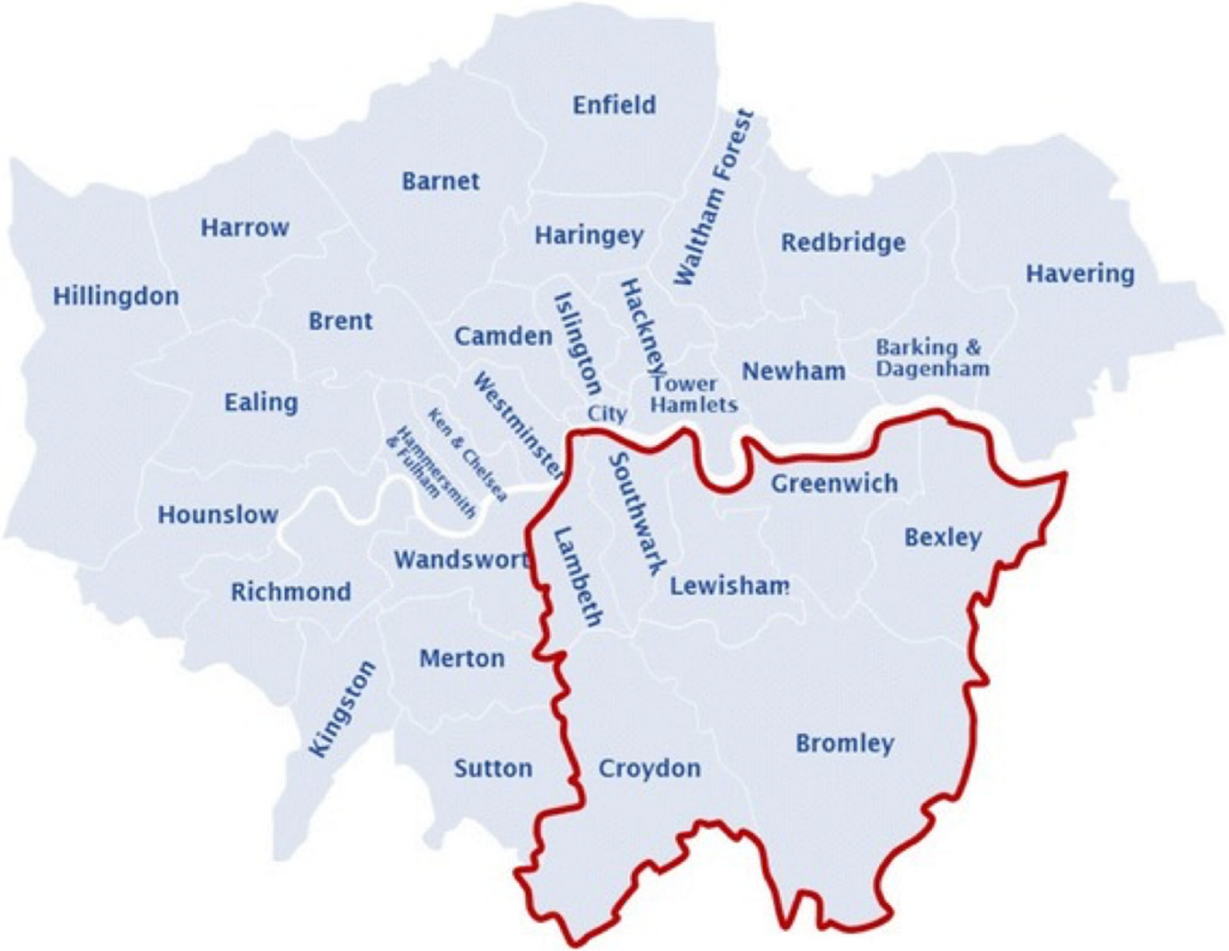
Catchment area of King’s Health Partners (KHP). Image adapted with permission from London City Council Government Directory [3].

King’s Health Partners (KHP) is an Academic Health Science Centre bringing together King’s College London, Guy’s and St Thomas’ NHS Foundation Trust (GSTT), King’s College Hospital NHS Foundation Trust and the South London and Maudsley NHS Foundation Trust, as shown in Fig. 1 [3].

The KHP Bladder Cancer Biobank was established in April 2017 as part of the overarching KHP Cancer Biobank. Bladder cancer patients are recruited from the Urology and Oncology departments of Guy’s Hospital and provide their consent to donate blood, urine, tissue surplus to diagnostic requirements and extra tissue samples just for the Biobank. Patients may be approached to give consent at any point of their treatment journey, therefore allowing the Biobank to access residual material from an earlier diagnosis. Thus, although patients only started giving their consent from June 2017, the Biobank has samples collected both before and after this date. Clinical data relevant to bladder cancer can be linked to the biobank samples and data across the entire patient treatment journey. The resource encourages translational research and provides a bridge to patient care. The Biobank samples can be used to exhaustion, creating new research data that can be re-used by investigators. In addition, there is a potential for patients of the KHP Bladder Cancer Biobank to be linked to our ongoing Trials within Cohort Study (TWiCs), the Graham Roberts Study [4], which provides a unique opportunity to answer a wide variety of research questions of a clinical, mechanistic, as well as supportive care nature in the area of bladder cancer.

The KHP Cancer Biobank is licenced by the Human Tissue Authority (reference number 12121) and is a NHS Research Ethics Committee approved Tissue Bank with generic ethical approval to supply bioresources (reference number 18/EE/0025) [5].

Because of our catchment area (Fig. 1), KHP covers a wide range of ethnic and socioeconomic backgrounds, hence providing a unique and valuable resource.

## Construction and content

**Fig. 2:**
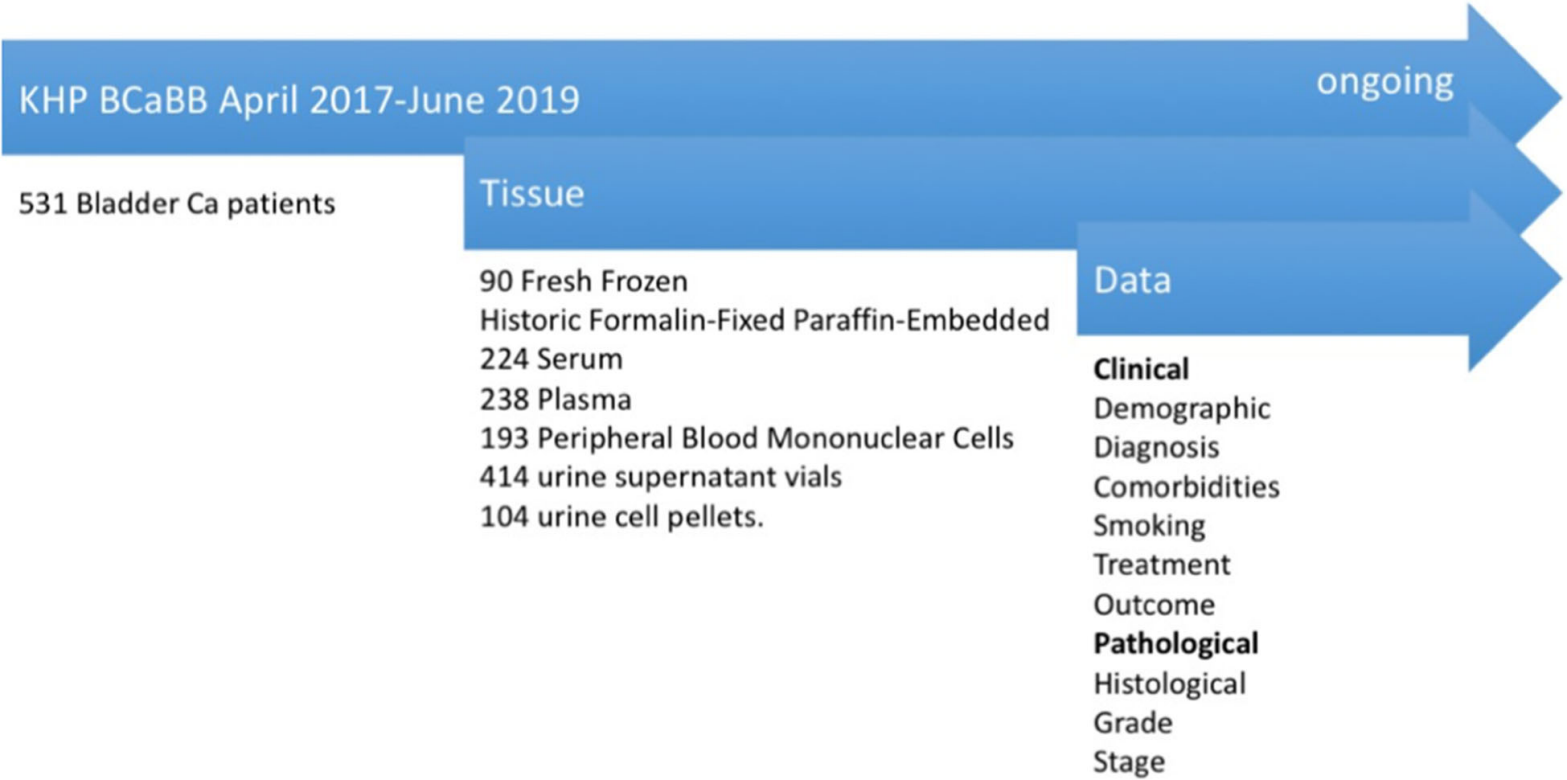
Data and tissue repository available in KHP Bladder Cancer biobank

A recent audit of the KHP Bladder Cancer Biobank highlighted that between April 2017–June 2019, 1089 patients were seen in a bladder clinic, with 997 subsequently diagnosed with bladder cancer. 552 patients gave their consent and following exclusion due to miscoding, 531 (53.3%) were included in the KHP Bladder Cancer Biobank. Recruitment for the biobank is ongoing (Fig. 2).

The patients who gave consent to the KHP Bladder Cancer Biobank can be considered representative of the total KHP bladder cancer population attending clinics at GSTT, therefore making the biobank an ideal platform to explore the different features of bladder cancer. The mean age of the overall bladder cancer population seen at GSTT between April 2017 and June 2019 was 70.9 years (SD: 11.6), as compared to 71.03 years (SD: 10.55) for the biobanked population (Table 1). In addition, we observed similarities in sex distribution (78.9% males in the consented population vs 74.9% in the total population) and tumour grade distribution (e.g. 62.0% Grade 3 in the consented population vs 59.6% in the total population) (Table 2).

**Table 1:** Demographic information of KHP Bladder Cancer Biobank from April 2017–June 2019

Variable	Consented (***n*** = 531)	%
*Age (years)*	*70.1 (SD: 11.6)*	
< 40	11	2.1
40–49	16	3.0
50–59	61	11.5
60–69	132	24.9
70–79	194	36.5
80–89	110	20.7
90–99	7	1.3
*Gender*		
Female	112	21.1
Male	419	78.9
*Ethnicity*		
British (White)	302	56.9
Any other White background	28	5.3
Mixed background	1	0.2
Asian background	8	1.5
Caribbean, African or Any other Black background	9	1.7
Any other Ethnic Group	5	0.9
Not Stated or Not Known	178	22.3
*Socioeconomic Status*		
Low	419	80.58
Middle	17	3.27
High	69	13.27
Missing	15	2.88
*Trial*		
Yes	32	6.0
No	499	94.0
*Comorbidities*		
Mean number of comorbidities	*3.02 (SD:1.82)*	
Hypertension	198	37.3
Diabetes	89	16.8
Other cancer	82	15.4
Hypercholesterolaemia	80	15.1
Respiratory disease	62	11.7
Cardiovascular disease	62	11.7
Chronic Kidney Disease	32	6.0
Psychiatric Disorder	23	4.3
*Smoking*		
Yes	183	34.5
No	33	6.2
Unknown	315	59.3

**Table 2:** Clinical information (diagnosis and pathological) of KHP Bladder Cancer Biobank from April 2017–June 2019

Clinical variable	Consented (***n*** = 531)	%
***Diagnosis***		
New	342	64.4
Recurrence	189	35.6
***Histology***		
Urothelial Cell Carcinoma	308	58.0
Papillary Urothelial Cell Carcinoma	165	31.1
Squamous Cell Carcinoma	17	3.2
Adenocarcinoma	7	1.3
Micropapillary variant	7	1.3
Plasmacytoid variant	3	0.6
Paraganglioma	4	0.8
Papilloma	1	0.2
Small cell carcinoma	3	0.6
Sarcomatoid	2	0.4
Unknown	8	1.5
***Grade***		
G1	38	7.2
G2	125	23.5
G3	329	62.0
Unknown	39	7.3
***T stage***		
Ta/ Tis	193	36.3
T1	133	25.0
T2	106	20.0
T3	65	12.2
T4	14	2.6
Unknown	20	3.8
***N stage***		
N0	485	91.3
N1	24	4.5
N2	22	4.1
***M stage***		
M0	502	94.5
M1	29	5.5
***Lymphovascular invasion***		
Yes	42	7.9
No	489	92.1
***TURBT***		
Yes	428	80.6
No	103	19.4
***Surgery***		
Yes	261	49.2
No	270	50.8
***Chemotherapy***		
Neoadjuvant	37	7.0
Adjuvant	30	5.6
Chemotherapy	20	3.8
Palliative	6	1.1
Neoadjuvant and adjuvant	2	0.4
None	436	82.1
***Radiotherapy***		
Yes	47	8.9
No	484	91.1
***Intravesical Therapy***		0.0
Yes	180	33.9
Di Stasi	76	14,3
BCG	62	11.7
Mitomycin	33	6.2
BCG and mitomycin in different occasions	7	1.3
Di Stasi and mitomycin in different occasions	1	0.2
Other	1	0.2
No	351	66.1
***Intravesical Therapy Failure***		
Yes	35	6.6
Poorly tolerated	10	1.9
No	6	1.1
Unknown	129	24.3
Not applicable	351	66.1
***Laser ablation***		
Yes	9	1.7
No	522	98.3
***Immunotherapy***		
Yes	17	3.2
No	514	96.8

### Tissue/ biospecimen repository

The KHP Bladder Cancer biobank includes fixed, paraffin embedded samples that are surplus to diagnostic requirement from all patients who gave consent and underwent a surgical procedure. Where possible, the Biobank collects fresh tumour and normal tissue from both resections and at biopsy. The Biobank has specialist Advanced Practitioners who review all potential specimens for suitability and are trained to partially dissect a fresh specimen and take samples for research but without impacting on the diagnostic integrity. Fresh frozen tissue is validated and stored at − 80 °C until required. All procedures are fully documented, and ischaemic times recorded, generating a specific sample-related data that can be provided to users of the resource.

During the 2-year audit period, the Biobank has collected fresh frozen tumour samples from 90/178 surgical procedures (of which 73 were a biopsy). Fixed, paraffin-embedded blocks are available from all 178 surgical procedures and if the Biobank so wished, it could request access to any tissue samples taken from the 531 patients who gave their consent.

Matched whole blood samples are also collected, from which a number of derivates are obtained including peripheral blood mononuclear cells (PBMC), plasma, serum and red blood cells. The derivatives are aliquoted, frozen and stored at − 80 °C. From urine samples, derivatives of urine supernatant and urine cell pellets are also generated and similarly stored. Similar to the tissue collection, data is recorded on processing procedures and times, number of derivatives and volume per aliquot.

Both blood and urine samples have been collected for 38 patients, which have been processed into 193 peripheral blood mononuclear cell vials; 238 plasma vials, 224 serum vials, 414 urine supernatant vials and 104 urine cell pellets.

A unique biobank identifier is automatically assigned to each new sample, which links to the donor. A second Research ID number is also created at the same time, which is used by researchers. Hence, this double anonymisation system provides the biobank with inbuilt security processes which protect patient data. Moreover, this ensures the collection of comprehensive data profiles as pathological data is linked to clinical patient data.

### Data repository

All the data obtained has been collected from either GSTT Electronic Patient Records or Cancer Information System, contributing to the demographic (Table 1) and clinical (Table 2) database of the Biobank.

An overview of demographic and clinic-pathological data collected for the Biobank (Table 3) is outlined below in more detail.

**Table 3:** Overview of demographic and clinical information collected

Basics	Sample size	997
	Patients consented	531
	Number of variables	21
	Involvement in clinical trial	Yes, No
Demographics	Age	
	Gender	Male, Female
	Ethnicity	
	Inclusion in Clinical Trial	Yes, No
	Comorbidities	Hypertension, Diabetes, Other cancer, Hypercholesterolaemia, Respiratory disease, Cardiovascular disease, Chronic Kidney Disease, Psychiatric Disorder
	Smoking	Yes, No, Unknown
Bladder cancer characteristics	Diagnosis	New, Recurrent
	Histology	Urothelial Cell Carcinoma, Papillary Urothelial Cell Carcinoma, Squamous Cell Carcinoma, Adenocarcinoma, Micropapillary variant, Plasmacytoid variant, Paraganglioma, Papilloma, Small cell carcinoma, Sarcomatoid, Unknown
	Grade	G1, G2, G3
	TNM stage	
	Lymphovascular invasion	Yes, No
	TURBT	Yes, No
	Surgery	Cystoprostatectomy, Cystectomy, Nephrouretectomy, Uretectomy, Nephrouretectomy and cystoprostatectomy, Cystectomy and ureterectomy, Diverticulectomy, Nephrouretectomy and cystectomy, Nephrouretectomy and partial cystectomy, Ureterostomy, Urethrectomy, Exenteration, None
	Chemotherapy	Neoadjuvant, Adjuvant, Chemotherapy, Palliative, Neoadjuvant and adjuvant, None
	Radiotherapy	Yes, No
	Intravesical therapy	Di Stasi, BCG, Mitomycin, BCG and mitomycin in different occasions, Di Stasi and mitomycin in different occasions, Other, None
	Intravesical therapy failure	Yes, No
	Laser ablation	Yes, No
	Immunotherapy	Yes, Poorly tolerated, Unknown, No, Not Applicable

### Demographics

Demographic information includes age, gender, ethnicity and postcode (Table 1). Currently, the cohort comprises of 62.1% White, 1.5% Asian, 1.7% Black, 1.1% Mixed or other ethnic groups. The ethnic background of 178 patients (33.5%) is not stated or not known. Postcode provides an opportunity to obtain information about the social deprivation index of the patients. We observed similarities in socioeconomic status (SES) between those who gave consent to the biobank and the GSTT total bladder cancer population (80.6% of low socioeconomic status in the consented population vs 82.6% in the total population).

In addition, baseline information of the biobanked patients also provides data on comorbidities and smoking history [6], as the latter is a known risk factor for bladder cancer (Table 2).

Finally, in the demographic database we also collect data about trial participation. Of the 531 patients in the biobank, 32 (6%) are currently also enrolled in a clinical trial.

### Clinical information

Diagnostic information includes whether the bladder cancer diagnosis was new or recurrent, tumour histology, Grade, TNM staging and lymphovascular invasion (Table 2). A total of 35.6% patients in the biobank had a recurrent disease. In terms of histology, 89.1% patients had urothelial cell carcinoma, of which 31% were papillary urothelial cell carcinoma. Further details can be found in Table 2 and, as described above, the biobank population is representative of total bladder cancer population.

In addition to diagnostic information, the Bladder Cancer Biobank also contains treatment data that can be linked to samples (Table 2). For instance, 49.2% of patients had undergone a radical cystectomy.

### Utility and discussion

The biobank provides a valuable resource for research. As an example, we have outlined one of the ongoing studies below.

#### Phenotyping γδ T cells in bladder cancer- results from a pilot study

A recent analysis of over 18,000 tumour transcriptomes found a γδ T cell transcription signature to be strongly associated with a positive prognosis in human cancers [7]. γδ T cells have also been implicated in responses to Bacillus Calmette-Guerin (BCG) therapy of non-muscle invasive bladder cancer [8, 9]. However, studies in muscle-invasive bladder cancer have been limited. This project, which was approved by the Bladder Biobank Access Committee, uses a grid culture method to expand and isolate lymphocytes from tumour and para-tumoural tissue samples selected by the Biobank from fresh cystectomy specimens. Matched blood and urine samples from bladder cancer patients are also collected by research nursing staff and processed by the biobank which supplies cell suspensions for downstream phenotyping. The presence and phenotype of γδ T cells in bladder cancer and their associations with clinical features and outcomes are currently being examined and preliminary data has already been presented [10].

An additional arm of the project examines matched urine and blood samples from patients undergoing Bacillus-Calmette Guerin therapy for non-muscle invasive bladder cancer and these samples are also processed through the Biobank. Thus far, results show a γδ T cell compartment in tumour and para-tumoural tissue with carcinoma in situ affecting γδ T cell representation in both areas. Para-tumoural tissue also shows a stage related relationship with an increased percentage of γδ T cells in para-tumoural tissue surrounding higher stage tumours.

### Strengths and weakness

The KHP Bladder Cancer Biobank is a novel and valuable resource. Its size and depth allow the linkage of biospecimens and clinical data across the patient treatment journey with multidisciplinary input from both oncology and urology specialties. As detailed, the biobank is largely representative of the overall bladder cancer population of South East London. Despite this, currently a large proportion of bladder cancer patients diagnosed or treated at Guy’s Hospital are not consented to the biobank; however, this provides a further valuable opportunity to increase the sample size and tissue resources in the future. The Biobank is a resource to be utilised and can be accessed by any academic or commercial establishment who has a valid research question. Potential applicants are encouraged to apply through the KHP Cancer Biobank website.

## Conclusions

The KHP Bladder Cancer Biobank has assembled a comprehensive data repository, which is demographically and clinically representative of the total bladder cancer population in the Guy’s and St Thomas Trust catchment area. With a rich data and tissue repository, the Biobank is a valuable resource for future translational research.

## Data Availability

The KHP Bladder Cancer Biobank welcomes requests from academic and commercial collaborators who have a valid health research request. Initially, an Access application form is completed which is reviewed by a dedicated Access committee within King’s Health Partners who approve or reject applications according to predefined criteria.. All requests are dealt within a timely manner, with a response received usually within 2 weeks. Once approved, relevant costings for the samples are drawn up and agreed with the collaborators. More information about the Biobank and application process can be found on: https://www.khpbiobank.co.uk/

## Abbreviations

BC: Bladder Cancer
KHP: King’s Health Partners
TWiC: Trials within cohort study
PBMC: Peripheral blood mononuclear cells
GSTT: Guy’s and St Thomas’ NHS Foundation Trust
SES: Socioeconomic Status
BCG: Bacillus Calmette-Guerin
